# Architecture of Physical, Cognitive, and Emotional Symptoms at Preseason Baseline in Adolescent Student Athletes With a History of Mental Health Problems

**DOI:** 10.3389/fneur.2020.00175

**Published:** 2020-03-20

**Authors:** Grant L. Iverson, Payton J. Jones, Justin E. Karr, Bruce Maxwell, Ross Zafonte, Paul D. Berkner, Richard J. McNally

**Affiliations:** ^1^Department of Physical Medicine and Rehabilitation, Harvard Medical School, Boston, MA, United States; ^2^Spaulding Rehabilitation Hospital and Spaulding Research Institute, Boston, MA, United States; ^3^Home Base, A Red Sox Foundation and Massachusetts General Hospital Program, Boston, MA, United States; ^4^MassGeneral Hospital for Children™ Sport Concussion Program, Boston, MA, United States; ^5^Department of Psychology, Harvard University, Cambridge, MA, United States; ^6^Department of Psychiatry, Harvard Medical School, Boston, MA, United States; ^7^Department of Computer Science, Colby College, Waterville, ME, United States; ^8^Department of Physical Medicine and Rehabilitation, Spaulding Rehabilitation Hospital, Massachusetts General Hospital, Brigham and Women's Hospital, Harvard Medical School, Home Base, A Red Sox Foundation and Massachusetts General Hospital Program, Boston, MA, United States; ^9^Health Services and the Department of Biology, Colby College, Waterville, ME, United States

**Keywords:** child, adolescent, concussion, mental health, sports

## Abstract

**Objective:** Pre-injury mental health problems are associated with greater symptom reporting following sport-related concussion. We applied a statistical and psychometric approach known as network analysis to examine the interrelationships among symptoms at baseline in adolescent student athletes with a history of mental health problems.

**Design:** Cross-sectional study.

**Setting:** High schools in Maine, USA.

**Participants:** A cohort of 44,527 adolescent student athletes completed baseline preseason testing with ImPACT® between 2009 and 2015, and those with a history of mental health problems reporting at least one symptom were included (*N* = 2,412; 14–18 years-old, 60.1% girls).

**Independent Variables:** Self-reported history of treatment for a psychiatric condition.

**Main Outcome Measures:** Physical, cognitive, and emotional symptoms from the Post-Concussion Symptom Scale.

**Results:** Student athletes reported high frequencies of emotional symptoms (nervousness: boys = 46.6%, girls = 58.3%; irritability: boys = 37.9%, girls = 46.9%; sadness: boys = 38.7%, girls = 53.2%), sleep/arousal-related symptoms (trouble falling asleep: boys = 50.4%, girls = 55.1%; sleeping less than usual: boys = 43.8%, girls = 45.2%; and fatigue: boys = 40.3%, girls = 45.2%), headaches (boys = 27.5%, girls = 41.8%), and inattention (boys = 47.8%, girls = 46.9%) before the start of the season. Although uncommonly endorsed, dizziness was the most central symptom (i.e., the symptom with the highest aggregate connectedness with different symptoms in the network), followed by feeling more emotional and feeling slowed down. Dizziness was related to physical and somatic symptoms (e.g., balance, headache, nausea, numbness/tingling) whereas increased emotionality was related to sadness, nervousness, and irritability. Feeling slowed down was connected to cognitive (e.g., fogginess, forgetfulness), and sensory symptoms (e.g., numbness/tingling, light sensitivity). There were no gender differences in the symptom network structure.

**Conclusions:** We examined the interconnections between symptoms reported by student athletes with mental health problems at preseason baseline, identifying how physical, cognitive, and emotional symptoms interact and potentially reinforce each other in the absence of injury. These findings are a step toward informing more precise interventions for this subgroup of athletes if they are slow to recover following concussion.

## Introduction

A history of treatment for mental health problems (1), particularly depression (2), heightens risk for poor clinical outcome among athletes who experience concussion (3). The mechanisms mediating this association remain unclear, but are likely multifactorial, related to dispositional characteristics, pre-injury symptom experience, the neurometabolic consequences of concussion, or a combination thereof. Hence, understanding how student athletes with prior mental health problems experience symptoms *before* a concussion can aid interpretation of symptoms in those with delayed recovery following a concussion. The interrelations among symptoms before injury might have implications for the experience of symptoms acutely, sub-acutely, and post-acutely following injury—and more importantly, understanding these interrelations may foster a more targeted approach to treatment and rehabilitation (4).

Network analysis is a statistical and psychometric method for estimating the interrelationships among symptoms (5–8). Network analysis has been used in a large number of studies in psychology and psychiatry (9), yielding important insights in depression, for example (10–14). Rather than conceptualizing depression as the underlying common cause of the symptoms that signal its presence, network theorists view depression as an emergent phenomenon, issuing from the dynamic interactions among its constituent features (e.g., insomnia, fatigue, anhedonia). Some features are core symptoms (e.g., sadness and anhedonia), whereas others are not (e.g., anxiety and sympathetic arousal) (12). Depression and anxiety commonly co-occur, and cross-sectional network analysis studies have illustrated *how* the symptoms of major depressive disorder and generalized anxiety disorder are interconnected and mutually amplifying (15–17).

Network analysis has never been applied to research relating to baseline testing of student athletes or outcome from sport-related concussion (18). A network approach may explain why self-reported symptoms co-occur rather than emerge solely or primarily from a latent common cause. Whereas, previous factor models of symptoms at preseason baseline have found multidimensionality of the symptom scales, these models attribute co-occurrence among symptoms to a latent factor, not to causal interactions among the symptoms themselves (e.g., cognitive-sensory, vestibular-somatic, sleep-arousal, and affective symptoms) (19). In contrast, network analysis estimates potential causal interactions among symptoms. Rather than latent constructs of (i) affective symptoms → nervousness, (ii) cognitive-sensory symptoms → difficulty concentrating, and (iii) cognitive-sensory symptoms → difficulty remembering, perhaps nervousness → difficulty concentrating → difficulty remembering. Adopting a network perspective in studies involving athletes at risk for slow recovery from concussion might help us identify single, paired, or small clusters of strongly interconnected symptoms that could be initial targets for treatment and rehabilitation (4). The purpose of this study is to examine the architecture of baseline preseason physical, cognitive, and emotional symptoms in adolescent student athletes who have a history of treatment for mental health problems.

## Methods

### Participants

Participants in this multi-year, cross-sectional, descriptive study were drawn from a cohort of adolescent student athletes (14–18 years-old) from Maine, USA who completed baseline preseason testing with the Immediate Post-Concussion Assessment and Cognitive Testing (ImPACT®) battery between 2009 and 2015 and reported no history of concussion in the past 6 months (*N* = 44,527). This study focused on the subsample of student athletes (6.8%) who reported a history of psychiatric treatment (*n* = 3,027). Participants who reported no history of psychiatric treatment (82.3%; *n* = 37,063) or did not respond to this question (10.0%; *n* = 4,437) were excluded. Participants further were excluded if they reported a history of a neurological condition, specifically meningitis, seizure disorder, or brain surgery (*n* = 121), reported a diagnosis of autism (*n* = 36), completed the assessment in a language other than English (*n* = 11), or reported no symptoms on the Post-Concussion Symptom Scale (*n* = 447). Participants who reported no symptoms were excluded because these individuals are not conceptually part of the population of interest. This resulted in a final sample of 2,412 participants. The mean age and rates of other self-reported health history variables are summarized in Table 1. Their sports played at the time of baseline testing are presented in Table 2.

**Table 1:** Characteristics of the student athletes with a history of mental health treatment.

		**Boys (*****n*** **=** **963)**		**Girls (*****n*** **=** **1,449)**	
		***n***	***%***	***n***	***%***
Age		*M* = 15.98	*SD* = 1.33	*M* = 15.76	*SD* = 1.19
ADHD diagnosis	Yes	412	42.8%	352	24.3%
	No	551	57.2%	1,097	75.7%
History of substance abuse treatment	Yes	59	6.1%	35	2.4%
	No	904	93.9%	1,414	97.6%
History of migraine treatment	Yes	134	13.9%	247	17.0%
	No	829	86.1%	1,202	83.0%
History of headache treatment	Yes	203	21.1%	345	23.8%
	No	760	78.9%	1,104	76.2%
Learning disability or dyslexia diagnosis	Yes	150	15.6%	152	10.5%
	No	813	84.4%	1,297	89.5%
Special education	Yes	127	13.2%	125	8.6%
	No	836	86.8%	1,324	91.4%
History of speech therapy	Yes	127	13.2%	127	8.8%
	No	836	86.8%	1,322	91.2%
Repeated academic grade	Yes	133	13.8%	109	7.5%
	No	830	86.2%	1,340	92.5%
Number of previous concussions	0	692	72.5%	1,121	78.0%
	1	139	14.6%	203	14.1%
	2	65	6.8%	72	5.0%
	3	33	3.5%	30	2.1%
	4	19	2.0%	7	0.5%
	≥5	7	0.7%	4	0.3%

**Table 2:** Primary sport affiliation by gender.

	**Boys (*****n*** **=** **963)**		**Girls (*****n*** **=** **1,449)**	
	***n***	***%***	***n***	***%***
Baseball/softball	33	3.4	77	5.3
Basketball	78	8.1	123	8.5
Cheerleading	2	0.2	254	17.5
Field hockey	0	0.0	197	13.6
American football	291	30.2	4	0.3
Ice hockey	56	5.8	29	2.0
Lacrosse	49	5.1	74	5.1
Skiing/snowboarding	27	2.8	37	2.6
Soccer	189	19.6	307	21.2
Swimming	16	1.7	46	3.2
Tennis	17	1.8	44	3.0
Track & field/cross country	98	10.2	117	8.1
Volleyball	0	0.0	55	3.8
Wrestling	41	4.3	9	0.6
Other	66	6.9	76	5.2

### Measures

A demographics and health history survey is embedded in ImPACT®, a computerized neuropsychological screening battery designed for use in sport concussion programs (20). The health history survey asks the student whether he or she has “had problems with ADD/hyperactivity” or been “diagnosed with ADHD” (attention-deficit hyperactivity disorder), been diagnosed with a learning disability or dyslexia, received special education services, or repeated a grade. These questions require a yes or no response. The health history survey also asks about the number of times the student has been diagnosed with a concussion as well as past treatment for headaches, migraines, epilepsy, brain surgery, meningitis, substance use, or a psychiatric condition (e.g., anxiety or depression). The question “treatment for a psychiatric condition (e.g., anxiety of depression)” was used to select subjects for this study. Youth who answered yes to this question were included.

The Post-Concussion Symptom Scale (21, 22) is a component of ImPACT®. This standardized self-report questionnaire includes 22 symptoms (see Table 3) that are rated from zero to six in terms of severity, with 1 or 2 reflecting “mild,” 3 or 4 reflecting “moderate,” and 5 or 6 reflecting “severe” problems with a given symptom. The internal consistency of the scale ranges from 0.88 to 0.94 in high school and college students, and 0.92 to 0.93 in concussed athletes (22). Students completed the Post-Concussion Symptom Scale independently.

**Table 3:** Percentage endorsing individual symptoms stratified by gender.

	**Boys** ***n*** **=** **963 (*****n*** **=** **1,183)**		**Girls** ***n*** **=** **1,449 (*****n*** **=** **1,676)**	
	**Mild or Greater**	**Moderate or Greater**	**Mild or Greater**	**Moderate or Greater**
Headache	27.5% (22.4%)	7.2% (5.8%)	41.8% (36.1%)	15.7% (13.5%)
Vomiting	6.4% (5.2%)	2.4% (1.9%)	5.9% (5.1%)	1.6% (1.4%)
Nausea	8.7% (7.1%)	2.7% (2.2%)	13.2% (11.4%)	4.3% (3.8%)
Balance problems	10.6% (8.6%)	1.8% (1.4%)	15.9% (13.8%)	4.5% (3.9%)
Dizziness	13.5% (11.0%)	3.7% (3.0%)	21.9% (19.0%)	6.1% (5.3%)
Trouble falling asleep	50.4% (41.0%)	25.8% (21.0%)	55.1% (47.6%)	31.7% (27.4%)
Fatigue	40.3% (32.8%)	16.9% (13.8%)	44.9% (38.8%)	23.1% (19.9%)
Sleeping more than usual	17.3% (14.1%)	7.9% (6.4%)	19.8% (17.1%)	10.4% (9.0%)
Sleeping less than usual	43.8% (35.7%)	23.3% (18.9%)	45.2% (39.1%)	24.8% (21.4%)
Sensitivity to light	22.4% (18.3%)	6.7% (5.5%)	26.8% (23.2%)	9.8% (8.5%)
Drowsiness	35.2% (28.7%)	11.9% (9.7%)	37.8% (32.7%)	15.5% (13.4%)
Sensitivity to noise	14.1% (11.5%)	4.5% (3.6%)	18.3% (15.8%)	6.6% (5.7%)
Irritability	37.9% (30.9%)	17.7% (14.4%)	46.9% (40.5%)	21.5% (18.6%)
Nervousness	46.6% (38.0%)	18.4% (15.0%)	58.3% (50.4%)	30.6% (26.5%)
Sadness	38.7% (31.5%)	18.5% (15.0%)	53.2% (46.0%)	27.6% (23.9%)
Feeling more emotional	32.9% (26.8%)	14.7% (12.0%)	55.7% (48.2%)	29.7% (25.7%)
Numbness or tingling	9.7% (7.9%)	2.9% (2.4%)	8.5% (7.3%)	2.9% (2.5%)
Feeling mentally foggy	21.9% (17.8%)	6.2% (5.1%)	20.6% (17.8%)	7.0% (6.0%)
Feeling slowed down	22.3% (18.2%)	8.5% (6.9%)	21.9% (19.0%)	8.4% (7.3%)
Difficulty concentrating	47.8% (38.9%)	20.5% (16.7%)	46.9% (40.5%)	21.5% (18.6%)
Difficulty remembering	24.9% (20.3%)	8.3% (6.8%)	21.3% (18.4%)	8.6% (7.5%)
Visual problems	15.1% (12.3%)	4.9% (4.0%)	17.3% (14.9%)	6.1% (5.3%)

*Note. The primary values in this table are from youth who reported previous treatment for a psychiatric condition and at least one symptom on the Post-Concussion Symptom Scale (boys: n = 963; girls: n = 1,449). The values provided in parentheses are the rates for larger samples of youth with a history of psychiatric treatment, including those who reported no symptoms on the Post-Concussion Symptom Scale (boys: n = 1,183; girls: n = 1,676). As presented above, symptom endorsement frequencies were higher after excluding those reporting no symptoms*.

### Statistical Analyses

#### Network Estimation

The network analyses were conducted in R (23) with the packages *qgraph* (24) and *glasso* (25, 26). We estimated a network whereby the *edges* (lines) connecting pairs of *nodes* (symptoms) signify partial correlations between symptoms, adjusting for the influence of all other symptoms in the network. Using the graphical LASSO (least absolute shrinkage and selection operator) algorithm (26), we regularized the network, thereby retaining the (“thickest”) edges having the largest magnitude and shrinking the trivially small, likely spurious ones to zero. The *qgraph* package provides an extended Bayesian information criterion (EBIC) model comparison routine that identifies the tuning parameter that optimizes model fit and parsimony (27), given a specific value of the hyperparameter gamma (γ). We used the default gamma value of 0.5 implemented in R to provide a balance between sensitivity and specificity. The process estimates 100 distinct models that vary in their sparsity, and the one with the lowest EBIC values is retained as the model that best maximizes the number of likely genuine edges while minimizing likely spurious ones (5). We used *qgraph* to create the graphs.

#### Centrality and Visualization

To identify the most highly connected (“central”) nodes, we computed the *expected influence centrality* metric for each node (28). In an undirected network, the expected influence of a given node (symptom) is the sum of all edge weights (lines) connected to it. Unlike the *strength centrality* metric, which sums the absolute values of the edges connected to a node irrespective of whether the edge signifies a positive or negative partial association, expected influence respects of the sign of edge before summing the edge values. Multidimensional scaling (MDS) (29) was used to create network plot layouts. In these layouts, distances between nodes roughly reflect edge weights. Specifically, MDS layouts place nodes with strong similarities (positive edge values) close together. The accuracy of an MDS layout can be assessed by using the reported *stress-1* value of the MDS fit (30). A small repulsion force was added to our MDS fit to avoid node overlap.

#### Gender Differences

Invariance between the networks of boys and girls was tested via the *networktree* approach (31, 32) which uses recursive-partitioning to search for optimal splits in pre-specified variables. For this analysis, we pre-specified gender as a potential split variable. The networktree approach searches for invariance in the correlation structure of the data. As a sensitivity check, we used the NetworkComparisonTest (33, 34) to perform tests on the global strength and network structure invariance of the network separated by gender.

#### Sensitivity Analyses

Centrality estimates can be affected if certain nodes (symptoms) have a restricted range of measurement or the variance between nodes differs (35). To test for this possibility, we computed correlations between strength centrality values and node variances. A positive correlation suggests the possible presence of this problem. Using strength centrality is ideal for this test because problems with inflated variance should influence edges whether positive or negative.

Another problem potentially distorting centrality metrics as well as violating a key assumption of network analysis (i.e., that the matrix is “positive definite”) occurs when multiple nodes measure the same construct (e.g., are synonyms for the same symptom). We tested for such node redundancy by the goldbricker function (36, 37), comparing topological overlap to assess for problematically overlapping nodes. That is, two nodes are deemed redundant if they bear the same relation to many other nodes in the network. We tested for pairs of nodes sharing topological overlap >25% with an alpha level of 0.01. In addition, we estimated an unregularized partial correlation network (i.e., a concentration network); we note any conflicting results between the networks in the Results section.

#### Network Stability

We assessed the replicability of our network by using the bootnet package to perform bootstrapping (5). Non-parametric bootstrapping generates confidence intervals for edge and centrality parameters. Non-parametric bootstrapping also facilitates direct comparisons across edges and centrality values. Confidence intervals and difference plots for edge and centrality parameters guided our interpretations. In addition, a case-dropping bootstrap enabled us to calculate a correlation-stability coefficient for the network. A correlation-stability coefficient signifies the maximum proportion of participants in the full sample that can be dropped while maintaining a high degree of correlation (r > 0.7) of centrality values with the full-sample network. The suggested benchmarks for stability area correlation-stability coefficient are 0.25 (adequate stability) or 0.5 (good stability).

## Results

Participants who reported symptoms vs. those who did not report any symptoms were compared on demographic characteristics and academic and health history. They did not differ in their age, number of self-reported concussions, or their frequency of reporting an ADHD diagnosis, learning disability diagnosis, repeating a grade, receiving speech therapy, or receiving treatment for a substance use disorder. Those participants reporting symptoms were more likely to be girls (χ^2^ = 13.42, *p* < 0.001), report a history of special education services (χ^2^ = 4.56, *p* = 0.033), and report previous treatment for headaches (χ^2^ = 23.51, *p* < 0.001), or migraines (χ^2^ = 7.56, *p* = 0.006).

The percentages of adolescent student athletes endorsing each symptom, stratified by gender and by symptom severity, are presented in Table 3. The sample used for analysis excluded participants who did not endorse any symptoms on the questionnaire, which lead to higher symptom endorsement frequencies than would be observed if participants reporting no symptoms were included. In Table 3, we also have included frequencies of symptom endorsement for the larger sample that includes those reporting no symptoms (*n* = 2,859), which shows the differences in endorsement frequencies based on their exclusion. Emotional symptoms, such as nervousness (boys = 46.6%, girls = 58.3%), irritability (boys = 37.9%, girls = 46.9%), and sadness (boys = 38.7%, girls = 53.2%) were endorsed frequently. Trouble falling asleep (boys = 50.4%, girls = 55.1%), sleeping less than usual (boys = 43.8%, girls = 45.2%), and fatigue (boys = 40.3%, girls = 45.2%) also were commonly endorsed. Approximately half of the sample endorsed difficulty with concentration (boys = 47.8%, girls = 46.9%), and a large minority endorsed headaches (boys = 27.5%, girls = 41.8%).

### Network Architecture of Symptoms

**Figure 1:**
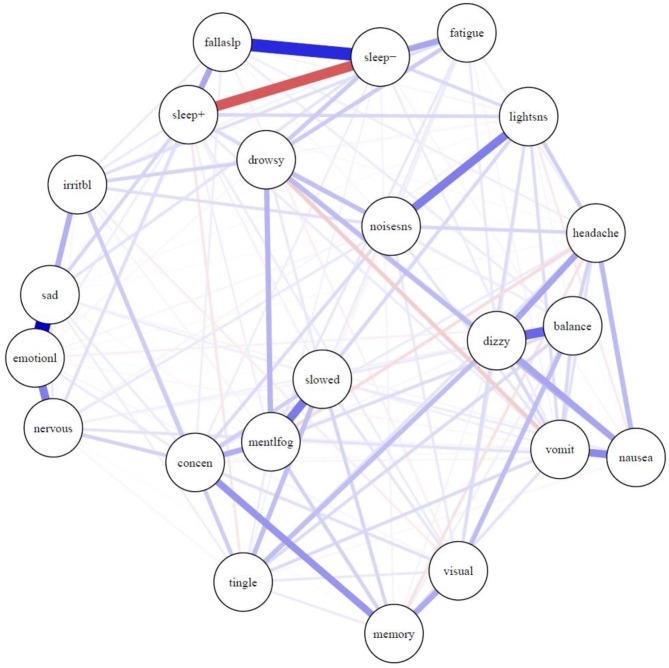
Network of symptoms during preseason baseline testing in student athletes with a history of mental health problems. Symptoms: balance, balance problems; concen, difficulty concentrating; dizzy, dizziness; drowsy, drowsiness; emotionl, feeling more emotional; fallaslp, trouble falling asleep; fatigue, fatigue; headache, headache; irritbl, irritability; lightsns, light sensitivity; memory, difficulty remembering; mentlfog, mentally foggy; nausea, nausea; nervous, nervousness; noisesns, noise sensitivity; sad, sadness; sleep-, sleeping less than usual; sleep+, sleeping more than usual; slowed, feeling slowed down; tingle, numbness and tingling; visual, visual problems; vomit, vomiting.

The network of symptoms is presented in Figure 1. Most connections between symptoms were positive, indicating that greater activation of one symptom is uniquely associated with greater activation of other symptoms. One exception is the negative correlation between sleeping more than usual (“sleep+”) and sleeping less than usual (“sleep–”), which is expected, and is illustrated in red. There was a strong relationship between sleeping less than usual (“sleep–”) and trouble falling asleep (“fallaslp”), and a fairly strong relationship between sleeping less than usual and fatigue. There was a very strong relationship between feeling more emotional (“emotionl”) and sadness (“sad”). There were relatively strong relationships between feeling more emotional (“emotionl”) and nervousness (“nervous”) and irritability (“irritbl”). There was a very strong association between sensitivity to light (“lightsns”) and sensitivity to noise (“noisesns”), between dizziness (“dizzy”) and balance problems (“balance”), and between feeling mentally foggy (“mentlfog”) and feeling slowed down (“slowed”). The *stress-1* for the MDS fit was 0.26 before adding the repulsion parameter. The unrepulsed network had a highly similar layout with some overlapping nodes.

### Centrality Analyses

**Figure 2:**
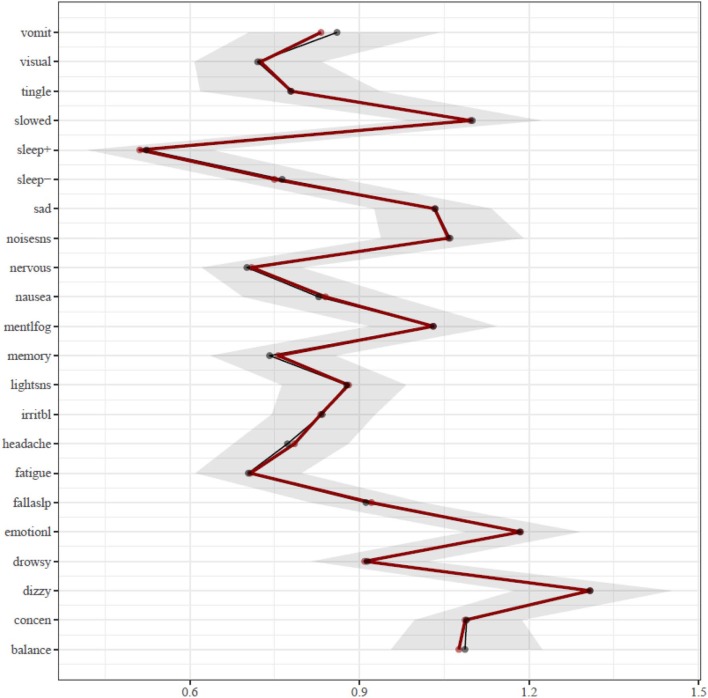
Expected influence centrality estimates. The bootstrap means are presented in black and the sample means are presented in red. Symptoms: balance, balance problems; concen, difficulty concentrating; dizzy, dizziness; drowsy, drowsiness; emotionl, feeling more emotional; fallaslp, trouble falling asleep; fatigue, fatigue; headache, headache; irritbl, irritability; lightsns, light sensitivity; memory, difficulty remembering; mentlfog, mentally foggy; nausea, nausea; nervous, nervousness; noisesns, noise sensitivity; sad, sadness; sleep−, sleeping less than usual; sleep+, sleeping more than usual; slowed, feeling slowed down; tingle, numbness and tingling; visual, visual problems; vomit, vomiting.

The results of the centrality analysis are presented in Figure 2. Centrality is calculated as the sum of the edge weights that connect a node (i.e., symptom) to other nodes in the network, meaning the most central symptoms have the strongest aggregate connections with other symptoms in the network. Hence, the activation of a symptom having high expected influence centrality statistically predicts the co-occurrence of other symptoms strongly associated with it. The most central symptoms in the network are dizziness (“dizzy”) and feeling more emotional (“emotionl”), followed by feeling slowed down (“slowed”). The centrality of these symptoms was significantly greater than most other symptoms in the network. The dizziness node shared most of its strong connections with other physical and somatic-type symptoms, including balance problems (“balance”), headache, nausea, and numbness and tingling (“tingle”). The feeling more emotional (“emotionl”) node shared its strongest connection with sadness (“sad”), nervousness (“nervous”), and irritability (“irritbl”). The feeling slowed down (“slowed”) node shared its strongest connection with feeling mentally foggy (“mentlfog”), and weaker associations with diverse symptoms such as numbness and tingling (“tingle”), light sensitivity (“lightsns”), and difficulty remembering (“memory”). The difficulty concentrating (“concen”) node has its strongest associations with difficulty remembering (“memory”) and feeling mentally foggy (“mentlfog”), but it also has diverse associations with numbness and tingling (“tingle”), feeling slowed down (“slowed”), irritability (“irritb”), nervousness (“nervous”), noise sensitivity (“noisesns”), and visual problems (“visual”).

### Gender Differences

Using a networktree approach, we found no significant differences in the structure of the symptoms between boys and girls. Moreover, the NetworkComparisonTest revealed no significant differences for global strength or network invariance.

### Sensitivity Analyses

The association between node variance and strength centrality was examined, and there was a weak relationship (*r* = 0.02), suggesting that restricted ranges or differences in node variances did not lead to biased centrality estimates. We also tested for problematic topological overlap in the network, and the goldbricker function revealed one potential overlapping node: feeling more emotional (“emotionl”) and sadness (“sad”), which had only 15% significant differences in topology. This may indicate that these two nodes should be combined (averaged) or that one of them should be dropped from the network, casting some doubt on the central importance of this symptom, given that connections may be due primarily to relationships with overlapping symptoms (nodes). Given its conceptual relevance to youth with a prior history of mental health problems, and its logical coherence with these other nodes (symptoms), we opted to preserve it in our interpretation of the results, although with some caution. An unregularized partial correlation network displayed consistent properties with the graphical LASSO network.

### Stability

The stability of the network in Figure 1 exceeded the threshold for “good” stability (0.5), and in fact reached the maximum tested stability threshold of 0.75. This indicates that at least 75% of the sample could be dropped, and the expected influence values from this subsample would correlate at *r* = 0.7 or more with the original sample. In addition to the positive results from the case-dropping bootstrap, the non-parametric bootstrap also evidenced good stability. Confidence intervals were tight around parameter estimates for centrality and edge weight. Difference tests between parameters also consistently showed significance when comparing nodes with high centrality to nodes with low centrality.

## Discussion

To our knowledge, this is the first study to apply network analysis to examine the architecture of baseline, preseason symptom reporting in student athletes. We selected a sample of particular interest—those with a prior history of treatment for mental health problems—because (i) it is known that they report more symptoms than the general population of student athletes at baseline (38); (ii) their pattern of symptom reporting can resemble the post-concussion syndrome at baseline, prior to sustaining a sport-related concussion (38); and (iii) prior mental health problems are a risk factor for worse outcome and experiencing persistent symptoms following sport-related concussion (1–3). When these “at-risk” adolescent student athletes are seen in multidisciplinary specialty clinics because they have persistent symptoms following concussion, clinicians can struggle to disentangle the extent to which those symptoms reflect pre-injury dispositional characteristics, ongoing neurobiologically-based symptoms associated with injury, an emotional response to a specific persistent symptom, such as headaches, a stress reaction due to being removed from sports and falling behind academically, or some combination of factors. Our network analysis provides information about the inter-relations among symptoms that differs from other statistical techniques, such as factor analysis (19, 39–41), and it identified pairs and clusters of strongly interconnected symptoms that reveal the nature of pre-injury symptoms in these young people which could inform a more precise approach to post-injury treatment and rehabilitation (4, 18).

As seen in Figure 1, there were strong and intuitive pairwise associations between certain symptoms, such as (i) trouble falling asleep and sleeping less than usual, (ii) dizziness and balance problems, (iii) sensitivity to light and sensitivity to noise, and (iv) poor concentration and trouble remembering. As seen in Table 3, the frequency by which the boys and girls endorsed those individual symptoms ranged from uncommon to common, as previously described in a past study (38), but less is known about how pairs of symptoms are associated. Knowing that these symptoms are associated, and co-occur before injury, can help clinicians better understand persistent or chronic symptom reporting post-injury in youth with a history of mental health problems.

Feeling more emotional was one of the most central and interconnected symptoms in the network (see Figure 2), with its strongest connections with the other emotional symptoms (see Figure 1)—nervousness, sadness, and irritability—as expected. These same symptoms tend to load on an emotional factor in factor analytic studies with student athletes (19, 41). This intuitive and expected finding aligns with previous research that has demonstrated heightened emotional symptoms in student athletes with pre-existing mental health problems (42) and illustrates the importance of these emotional symptoms based on their frequency of endorsement and centrality in the symptom network. It is reasonable to speculate that treatment designed to reduce emotional difficulties might dampen and reduce other symptoms in the broader network, given their interconnectedness. However, this would need to be examined in youth with persistent symptoms following a concussion (and not assume it to be true based on the nature of pre-injury symptom reporting).

Interestingly, one of the most central and important symptoms in the network was dizziness, and it was strongly connected to other physical-somatic symptoms such as balance problems, headache, nausea, and numbness and tingling. Physical symptoms tend to cluster and co-occur in factor analytic studies, but these symptoms are often distributed across two factors, such as neurocognitive and somatic (41) or cognitive-migraine-fatigue and somatic (19). Dizziness was only endorsed by 13.5% of boys and 21.9% of girls, which might be interpreted by some as prima facie evidence of that symptom not being as important as others that are endorsed with greater frequency. However, dizziness is a very central symptom with connections to multiple other symptoms, illustrating how physical post-concussion-like symptoms are inter-related, and potentially mutually reinforcing, in the absence of injury—and thus they could reflect emotional problems in some youth who have chronic symptoms following an injury.

Another interesting finding was the absence of gender differences in the network architecture of symptoms. It is well-established in the literature that girls and young women report more symptoms than boys and young men during preseason evaluations (19, 22, 43). In this sample of boys and girls with a history of treatment for mental health problems, the girls had greater symptom reporting but the network interrelationships among those symptoms did not differ from that of the boys. This suggests that how symptoms are inter-related in boys and girls with prior mental health problems is similar. Future researchers need to determine whether there are gender differences in the network structure of symptoms in subgroups of youth who are slow to recover from a sport-related concussion.

This study provides some initial insight into symptom networks among student athletes at-risk for prolonged recovery following concussion, but also has several methodological limitations. This study focused on student athletes with a history of mental health problems, which was defined based on self-reported history of psychiatric treatment. Although student athletes who undergo repeated baseline testing are consistent in reporting a history of clinical conditions [44], this method does not allow for any specificity in regard to the what psychiatric condition may have been treated, who diagnosed a psychiatric condition, and the accuracy of any psychiatric diagnosis. Of note, this variable is embedded within the ImPACT® battery, and although non-specific, the question itself reflects widespread practice in baseline assessments of student athletes. This study also involves only a single measurement occasion and does not clarify the stability of a symptom network over time or after a sport-related concussion. Lastly, the study only involved analysis of the 22 items from the Post-Concussion Symptom Scale, which is a commonly used measure of symptom assessment in clinical practice, but may exclude relevant variables that could have an influence on the symptom network (e.g., perceived life stress, other psychiatric symptoms). This limitation is characteristic of many studies on student athletes, and future large-scale data collection efforts may benefit from thoughtful inclusion of additional variables that may contribute to a symptom network.

Additional research using network analyses could advance knowledge relating to symptom reporting in student athletes both before and after a concussion, and potentially inform precision rehabilitation for those who have specific clusters of symptoms, are slow to recover, or who have symptoms that persist for months (18). It would be useful to examine baseline symptom reporting with network analysis in other populations of student athletes with pre-existing developmental problems, such as ADHD, or health problems, such as migraine, because both are associated with greater baseline symptoms in general (38) and can complicate recovery, return to school, and return to sports following injury (3). Moreover, it would be of considerable interest to examine the stability of symptom networks in youth with pre-existing conditions, such as prior mental health problems, ADHD, or migraines, by comparing their symptom architecture over a 1-year or 2-years test-retest interval. This would help determine the extent to which the interrelations among symptoms appear stable. Finally, and most importantly, it will be important to apply this methodology to clarify how symptom reporting in concussed athletes varies across different time points following injury. Acutely following injury there is the potential to use this methodology for prognostic reasons. During the sub-acute and post-acute time periods, this methodology might be helpful for understanding the inter-relations among symptoms, especially in subgroups of athletes with more specific prominent symptoms, such as visual-vestibular difficulties or more pronounced emotional problems. By better understanding the architecture of symptom reporting in student athletes before and after sustaining a concussion, we might be able to better design more targeted and effective treatment and rehabilitation strategies for them.

## Data Availability Statement

Statistical analyses and outputs of all data and results used in this article will be made available by the authors, without undue reservation, to any qualified researcher.

## Ethics Statement

This project has been reviewed by the IRB at Colby College. The project meets all of the necessary criteria for human subject research under 45 CFR 46.111, including confidentiality and informed consent. This approval is provided under assurance IRB 00008881.

## Author Contributions

GI conceptualized the study, drafted several parts of the manuscript, and approved the final manuscript. PJ conducted the statistical analyses, helped draft the manuscript, critically reviewed the manuscript, and approved the final manuscript. JK assisted with some of the statistical analyses, critically reviewed the manuscript, and approved the final manuscript. BM helped design and coordinate data collection, managed the database of participants, and approved the final manuscript. RZ reviewed/revised the manuscript and approved the final manuscript. PB helped design and coordinate data collection, wrote the IRB, conceptualized the overall project, and edited/approved the final manuscript. RM helped conceptualize the study and the statistical analyses, helped draft the manuscript, and approved the final manuscript. All authors approved the final manuscript as submitted and agree to be accountable for all aspects of the work.

### Conflict of Interest

PB acknowledges funding for the Maine Concussion Management Initiative from the Goldfarb Center for Public Policy and Civic Engagement/Colby College and the Bill and Joan Alfond Foundation. GI has a clinical and consulting practice in forensic neuropsychology, including expert testimony, involving individuals who have sustained mild TBIs (including athletes). He has received research funding from several test publishing companies, including ImPACT Applications, Inc., CNS Vital Signs, and Psychological Assessment Resources (PAR, Inc.). He serves as a scientific advisor for Sway Operations, LLC, Highmark, Inc., and BioDirection, Inc. RZ and GI have received salary support from the Harvard Integrated Program to Protect and Improve the Health of National Football League Players Association Members. RZ acknowledges unrestricted philanthropic support from the Heinz Family Foundation. RZ serves on the Scientific Advisory Board of Myomo, Oxeia Pharma, and ElMInda. None of the authors nor their family members have a commercial or proprietary interest in ImPACT^®^. The remaining authors declare that the research was conducted in the absence of any commercial or financial relationships that could be construed as a potential conflict of interest.

## References

[1] Morgan CD Zuckerman SL Lee YM King L Beaird S Sills AK. Predictors of postconcussion syndrome after sports-related concussion in young athletes: a matched case-control study. J Neurosurg Pediatr. (2015) 15:589–98. 10.3171/2014.10.PEDS14356 25745949

[2] Zemek R Barrowman N Freedman SB Gravel J Gagnon I McGahern C. Clinical risk score for persistent postconcussion symptoms among children with acute concussion in the ED. JAMA. (2016) 315:1014–25. 10.1001/jama.2016.1203 26954410

[3] Iverson GL Gardner AJ Terry DP Ponsford JL Sills AK Broshek DK. Predictors of clinical recovery from concussion: a systematic review. Br J Sports Med. (2017) 51:941–8. 10.1136/bjsports-2017-097729 28566342 PMC5466929

[4] Collins MW Kontos AP Okonkwo DO Almquist J Bailes J Barisa M. Statements of agreement from the targeted evaluation and active management (team) approaches to treating concussion meeting held in pittsburgh, october 15-16, 2015. Neurosurgery. (2016) 79:912–29. 10.1227/NEU.0000000000001447 27741219 PMC5119544

[5] Epskamp S Fried EI. A tutorial on regularized partial correlation networks. Psychol Methods. (2018) 23:617–34. 10.1037/met0000167 29595293

[6] Dalege J Borsboom D van Harreveld F van der Maas HLJ. Network analysis on attitudes: a brief tutorial. Soc Psychol Personal Sci. (2017) 8:528–37. 10.1177/1948550617709827 28919944 PMC5582642

[7] Borsboom D Rhemtulla M Cramer AO van der Maas HL Scheffer M Dolan CV. Kinds versus continua: a review of psychometric approaches to uncover the structure of psychiatric constructs. Psychol Med. (2016) 46:1567–79. 10.1017/S0033291715001944 26997244

[8] Borsboom D Cramer AO. Network analysis: an integrative approach to the structure of psychopathology. Annu Rev Clin Psychol. (2013) 9:91–121. 10.1146/annurev-clinpsy-050212-185608 23537483

[9] Fried EI van Borkulo CD Cramer AO Boschloo L Schoevers RA Borsboom D. Mental disorders as networks of problems: a review of recent insights. Soc Psychiatry Psychiatr Epidemiol. (2017) 52:1–10. 10.1007/s00127-016-1319-z 27921134 PMC5226976

[10] van Loo HM van Borkulo CD Peterson RE Fried EI Aggen SH Borsboom D. Robust symptom networks in recurrent major depression across different levels of genetic and environmental risk. J Affect Disord. (2018) 227:313–22. 10.1016/j.jad.2017.10.038 29132074 PMC5815316

[11] Cramer AO van Borkulo CD Giltay EJ van der Maas HL Kendler KS Scheffer M. Major depression as a complex dynamic system. PLoS ONE. (2016) 11:e0167490. 10.1371/journal.pone.0167490 27930698 PMC5145163

[12] Fried EI Epskamp S Nesse RM Tuerlinckx F Borsboom D. What are 'good' depression symptoms? Comparing the centrality of DSM and non-DSM symptoms of depression in a network analysis. J Affect Disord. (2016) 189:314–20. 10.1016/j.jad.2015.09.005 26458184

[13] van Borkulo C Boschloo L Borsboom D Penninx BW Waldorp LJ Schoevers RA. Association of symptom network structure with the course of [corrected] depression. JAMA Psychiatry. (2015) 72:1219–26. 10.1001/jamapsychiatry.2015.2079 26561400

[14] Cramer AO Borsboom D Aggen SH Kendler KS. The pathoplasticity of dysphoric episodes: differential impact of stressful life events on the pattern of depressive symptom inter-correlations. Psychol Med. (2012) 42:957–65. 10.1017/S003329171100211X 22093641 PMC3315770

[15] Cramer AO Waldorp LJ van der Maas HL Borsboom D. Comorbidity: a network perspective. Behav Brain Sci. (2010) 33:137–50; discussion 50–93. 10.1017/S0140525X09991567 20584369

[16] Beard C Millner AJ Forgeard MJ Fried EI Hsu KJ Treadway MT. Network analysis of depression and anxiety symptom relationships in a psychiatric sample. Psychol Med. (2016) 46:3359–69. 10.1017/S0033291716002300 27623748 PMC5430082

[17] Curtiss J Klemanski DH. Taxonicity and network structure of generalized anxiety disorder and major depressive disorder: an admixture analysis and complex network analysis. J Affect Disord. (2016) 199:99–105. 10.1016/j.jad.2016.04.007 27100054

[18] Iverson GL. Network analysis and precision rehabilitation for the post-concussion syndrome. Front Neurol. (2019) 10:489. 10.3389/fneur.2019.00489 31191426 PMC6548833

[19] Kontos AP Elbin RJ Schatz P Covassin T Henry L Pardini J. A revised factor structure for the post-concussion symptom scale: baseline and postconcussion factors. Am J Sports Med. (2012) 40:2375–84. 10.1177/0363546512455400 22904209

[20] ImPACT Applications Inc Immediate Post-Concussion Assessment and Cognitive Testing (ImPACT®) Test: Technical Manual. Pittsburgh, PA: ImPACT Applications, Inc (2011).

[21] Lovell MR Collins MW. Neuropsychological assessment of the college football player. J Head Trauma Rehabil. (1998) 13:9–26. 10.1097/00001199-199804000-00004 9575253

[22] Lovell MR Iverson GL Collins MW Podell K Johnston KM Pardini D. Measurement of symptoms following sports-related concussion: reliability and normative data for the post-concussion scale. Appl Neuropsychol. (2006) 13:166–74. 10.1207/s15324826an1303_4 17361669

[23] R Core Development Team Editor. R: A Language and Environment for Statistical Computing. 3.01. Vienna: R Foundation for Statistical Computing (2012). Available online at: https://www.R-project.org/ (accessed July 25, 2019).

[24] Epskamp S Cramer AO Waldorp LJ Schmittmann VD Borsboom D qgraph: Network visualizations of relationships in psychometric data. J Stat Softw. (2012) 48:1–18. 10.18637/jss.v048.i04 (accessed July 25, 2019).

[25] Friedman J Hastie T Tibshirani R Graphical Lasso -Estimation Of Gaussian Graphical Models. (2014). Available online at: https://www.-stat.stanford.edu/~tibs/glasso

[26] Friedman J Hastie T Tibshirani R. Sparse inverse covariance estimation with the graphical lasso. Biostatistics. (2008) 9:432–41. 10.1093/biostatistics/kxm045 18079126 PMC3019769

[27] Chen J Chen Z Extended bayesian information criteria for model selection with large model spaces. Biometrika. (2008) 95:759–71. 10.1093/biomet/asn034

[28] Robinaugh DJ Millner AJ McNally RJ. Identifying highly influential nodes in the complicated grief network. J Abnorm Psychol. (2016) 125:747–57. 10.1037/abn0000181 27505622 PMC5060093

[29] Jones PJ Mair P McNally RJ. Visualizing psychological networks: a tutorial in R. Front Psychol. (2018) 9:1742. 10.3389/fpsyg.2018.01742 30283387 PMC6156459

[30] Mair P Borg I Rusch T. Goodness-of-fit assessment in multidimensional scaling and unfolding. Multivariate Behav Res. (2016) 51:772–89. 10.1080/00273171.2016.1235966 27802073

[31] Jones PJ Simon T Zeileis A Networktree: Recursice Partitioning of Network Models. R package version 0.2.2 (2019). Available online at: https://CRAN.R-project.org/package=networktree (accessed July 25, 2019).

[32] Jones PJ Mair P Simon T Zeileis A Network Model Trees. (2019). 10.31219/osf.io/ha4cw

[33] van Borkulo CD Boschloo L Kossakowski J Tio P Schoevers RA Borsboom D Comparing network structures on three aspects: a permutation test. Manuscript submitted for publication (2017). 10.13140/RG.2.2.29455.38569 35404628

[34] van Borkulo CD Epskamp S Jones PJ NetworkComparisonTest: Statistical Comparison of Two Networks Based on Three Invariance Measures. R package version 2.2.1 (2019). Available online at: https://cran.r-project.org/package=NetworkComparisonTest (accessed July 25, 2019).

[35] Terluin B de Boer MR de Vet HC. Differences in connection strength between mental symptoms might be explained by differences in variance: reanalysis of network data did not confirm staging. PLoS ONE. (2016) 11:e0155205. 10.1371/journal.pone.0155205 27880771 PMC5120783

[36] Levinson CA Brosof LC Vanzhula I Christian C Jones P Rodebaugh TL. Social anxiety and eating disorder comorbidity and underlying vulnerabilities: using network analysis to conceptualize comorbidity. Int J Eat Disord. (2018) 51:693–709. 10.1002/eat.22890 30102777

[37] Jones PJ Networktools: Tools for Identifying Important Nodes in Networks. R package version 1.2.0. (2018) 10.1155/2018/8268436

[38] Iverson GL Silverberg ND Mannix R Maxwell BA Atkins JE Zafonte R. Factors associated with concussion-like symptom reporting in high school athletes. JAMA Pediatr. (2015) 169:1132–40. 10.1001/jamapediatrics.2015.2374 26457403 PMC5333772

[39] Piland SG Motl RW Ferrara MS Peterson CL. Evidence for the factorial and construct validity of a self-report concussion symptoms scale. J Athl Train. (2003) 38:104–12. 12937520 PMC164898

[40] Piland SG Motl RW Guskiewicz KM McCrea M Ferrara MS. Structural validity of a self-report concussion-related symptom scale. Med Sci Sports Exerc. (2006) 38:27–32. 10.1249/01.mss.0000183186.98212.d5 16394950

[41] Joyce AS Labella CR Carl RL Lai JS Zelko FA. the postconcussion symptom scale: utility of a three-factor structure. Med Sci Sports Exerc. (2015) 47:1119–23. 10.1249/MSS.0000000000000534 25268538

[42] Brown DA Elsass JA Miller AJ Reed LE Reneker JC. Differences in symptom reporting between males and females at baseline and after a sports-related concussion: a systematic review and meta-analysis. Sports Med. (2015) 45:1027–40. 10.1007/s40279-015-0335-6 25971368

[43] Wojtowicz M Iverson GL Silverberg ND Mannix R Zafonte R Maxwell B. Consistency of self-reported concussion history in adolescent athletes. J Neurotrauma. (2017) 34:322–7. 10.1089/neu.2016.4412 27349296 PMC5335743

